# Connecting patients, practitioners, and regulators in supporting positive experiences and processes of shared decision making: A progress report

**DOI:** 10.1111/jep.13279

**Published:** 2019-10-09

**Authors:** Fiona Browne, Steven Bettles, Stacey Clift, Tim Walker

**Affiliations:** ^1^ Professional Standards Department General Osteopathic Council London UK

**Keywords:** communication, evaluation, osteopathy, patients, regulation, shared decision making, values, what matters

## Abstract

This paper describes a novel approach to explore how regulators, working with patients and practitioners, may contribute to supporting person‐centred care and processes of shared decision making in implementing professional standards and reducing harms. Osteopathic patients report high levels of patient care. However, areas of consultations less likely to be rated as high included “fully understanding your concerns,” “helping you to take control,” and “making a plan of action with you,” suggestive of a paternalistic approach to care and a barrier to the effective implementation of standards. This programme explored how to support patients and practitioners to make more explicit what is important to support consultations with better communication in accordance with standards. A series of workshops took place involving approximately 80 participants, which explored and identified practitioner and patient values; these were themed to develop a common framework and tested using case studies. Aspects of what enables or presents a barrier to a positive consultation were further explored with real patient narratives, and a range of resources were subsequently developed, which may support patients and practitioners to make explicit what is important to them in a consultation. A series of approaches and tools were then developed for piloting including patient curriculum vitae; patient goal planner; patient animation to support preparation for an appointment; infographic: a patient poster or leaflet; practitioner reflective tool; and an audio recording to increase awareness and understanding of values‐based practice. In conclusion, a range of approaches may help to support patients and practitioners to make explicit what is important to them in a consultation. The next phase of our programme will use a range of methods including cluster sampling, pre‐testing and post‐testing with the Consultation and Relational Empathy (CARE) measure tool, and interviews and focus groups with users and practitioners to demonstrate impact.

## 1 INTRODUCTION

Health professional regulators should seek to reduce harm, and this includes promoting positive clinical consultations. Contemporary health professional regulation should involve regulators exploring “through their interventions and influence, [how to] reduce the prevalence of instances of noncompliance with their standards” and “the creative use of existing mechanisms for the reduction of harm and supports professionalism and a joined‐up approach to regulation.”^1^ Although regulation has traditionally been perceived as taking action only after harm occurs, it is increasingly recognized that such an approach is less effective in reducing harm for patients generally and that regulators should work within their context to reduce harm and indeed embed standards.

A key challenge within the osteopathic context where practitioners work largely in sole practice and without employers or teams is the potential for development of diverse professional values. For example, evidence exists that professionalism is a key aspect of the delivery of health care to meet patient and public expectations.^2^ But we can also see that different professional groups in different contexts may hold different views about professionalism or professional behaviours.^3^

A further challenge in osteopathy is in improving patient agency within generally high satisfaction ratings. Osteopathic patients report very high levels of patient care, with up to 95% satisfaction.^4^ However, some areas of consultations were less likely to be rated as high as others, including “fully understanding your concerns”; “helping you to take control”; and “making a plan of action with you.”^4^ It would be possible for a practitioner to regard a high approval rating to indicate that they were meeting patient expectations and needs fully, but this may not be the case, as the findings are suggestive of a potentially paternalistic approach to care.

In 2015, the Supreme Court decided the case of *Montgomery v Lanarkshire Health Board*, which required practitioners to focus on dialogue in order to enable a patient to consent to treatment.^5^ This is discussed further below. Essentially, the Montgomery case changed the law to reflect what was already a requirement of many of the health care professions' standards of practice, including the General Osteopathic Council's (GOsC's) Osteopathic Practice Standards.^6^ There was, therefore, a legal as well as a professional requirement to understand what was important to a patient in order to provide the information needed by them to inform their decision making regarding their health care, and we were keen to explore this and support registrants in meeting these standards.

Analysis of complaints and concerns in relation to osteopathy shows that areas of communication and consent are a key theme raised by patients.^7^ There is evidence also that many concerns brought to the regulator did not result in regulatory sanctions.^8^ Complaints made to the regulator, which do not result in a sanction, suggest that there were no fitness‐to‐practise implications and that, therefore, the complaints should have been resolved in a different way and perhaps that better communication at the outset would have resolved such concerns at an earlier stage.

This paper aims to show how the GOsC, the statutory regulator for osteopaths in the United Kingdom, worked together with patients and practitioners and a fellow regulator, the General Dental Council, with similar goals including embedding standards, enhancing communication, and reducing harms. This work was undertaken in partnership with the Collaborating Centre for Values‐based Practice in Health and Social Care and the University College of Osteopathy and involved exploring how to support patients and practitioners to make explicit what is important to them to enhance communication and lead to a positive consultation and also to reduce harms that may be caused from miscommunication.

Such an approach involved a creative, agile, and innovative exploration of the role of the regulator in reducing harm. But this was not an academic study within a formal research context. It represents a pragmatic regulatory approach, aimed at exploring some key issues with stakeholders in order to better understand values in practice and to develop resources to support better communication and dialogue in shared decision making between practitioners and patients and the implementation of professional standards. Ethical approval was not sought or necessary for this programme of work.

## 2 THE WORKSHOPS

### 2.1 Workshop 1

In 2014, a workshop was designed and delivered by the GOsC, the Collaborating Centre for Values‐based Practice and the University College of Osteopathy. Attendees included osteopaths, patients, and other health professionals and regulators. Attendees were invited from across the health professional and regulatory sector to ensure a wide range of representation and views: representatives from the professional body, the Institute of Osteopathy, the Council of Osteopathic Educational Institutions, the Osteopathic Alliance (a group of osteopathic postgraduate institutions), students, the Professional Standards Authority, the Health and Care Professions Council, the General Medical Council, the academic and research community, and others. Patients also attended and were recruited following an invitation to the Private Patients Forum and via osteopaths in *The Osteopath* magazine. These expert stakeholders supported us to achieve the aims of the workshop, which were as follows:

- to understand the context in which osteopathy is practised;
- to understand how values come into osteopathic health care;
- to explore, debate, and discuss professional values in osteopathy through case studies, exercises, and other formats;
- to consider the relationship of values to professional standards and practice; and
- to explore next steps in supporting the embedding of standards.

**Figures 1:**
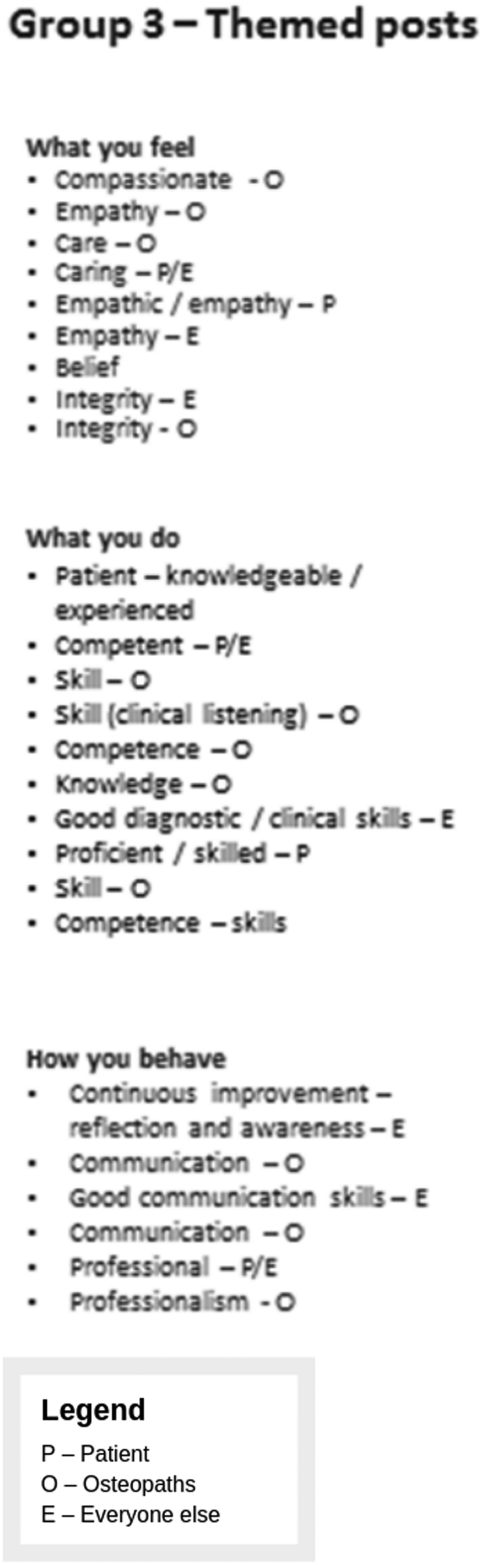
Example 1 of values identified by participants in the First Values Workshop held on November 2014

**Figure 2:**
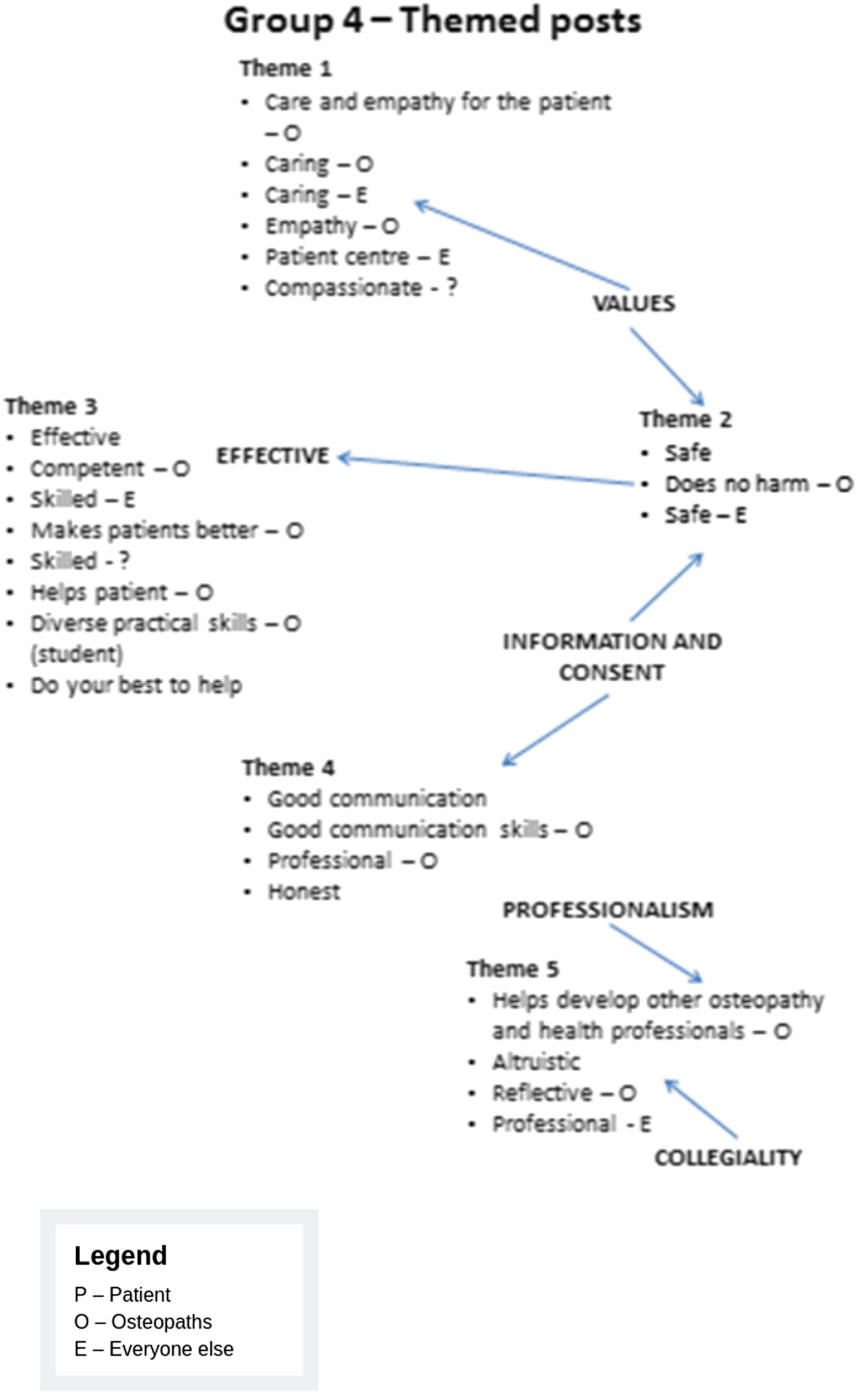
Example 2 of values identified by participants in the First Values Workshop held on November 2014

Participants were invited to identify their values for osteopathic care. Figures 1 and 2 provide some examples of the types of values that participants identified.

The outcome of the initial session was to identify that most people had different values but that they were not necessarily incompatible.

Next, participants were invited to undertake a “forced choice” exercise. Participants were given a scenario: “You develop the warning signs of a very serious illness. There are two options for treatment. Option 1 is a medicine which will either cure you completely or kill you immediately. Option 2 is a medicine which will give you a known period of remission and then you will die.”

Participants were asked to identify their preferred option and to identify the factors that were important to them in making the decision. What was clear was that there was a diverse range of views.^9^

Factors, such as age, approach to risk, family, and life circumstances, all contributed to decisions. The learning point was that individual preferences and values may lead to different decisions, even when the evidence supporting particular interventions is presented. Even though individuals may think their values are not controversial, others may apply them very differently in different contexts, and understanding the factors that contribute to decision making can be very important.

Next, participants were asked to theme their values and test them out in case studies.

The outcome of this workshop was to identify that professional identity and decision making were a strong value that influenced dialogue. It became clear that in applying values to decision making and case studies, there were clear tensions to be managed.

The findings from this workshop were analysed by our academic contributors from the University College of Osteopathy and St Catherine's College, Oxford, using thematic analysis and discussion to make sense of the themes identified from each group and also to develop a Common Core Values Framework (the framework) and also Component Values (subcategories of Core Values). This work took into account the detailed findings from the workshop, in order to develop a mechanism for exploring values in subsequent case studies. It was intended that the framework set out “what matters” in good osteopathic practice. The values could be seen to both describe how and why osteopaths practise and what patients and the wider public expect of good osteopathic practice. In a given situation, the values could be used as signposts for reflecting specifically on what constitutes good osteopathic practice from the particular perspectives of the people involved.

**Figure 3:**
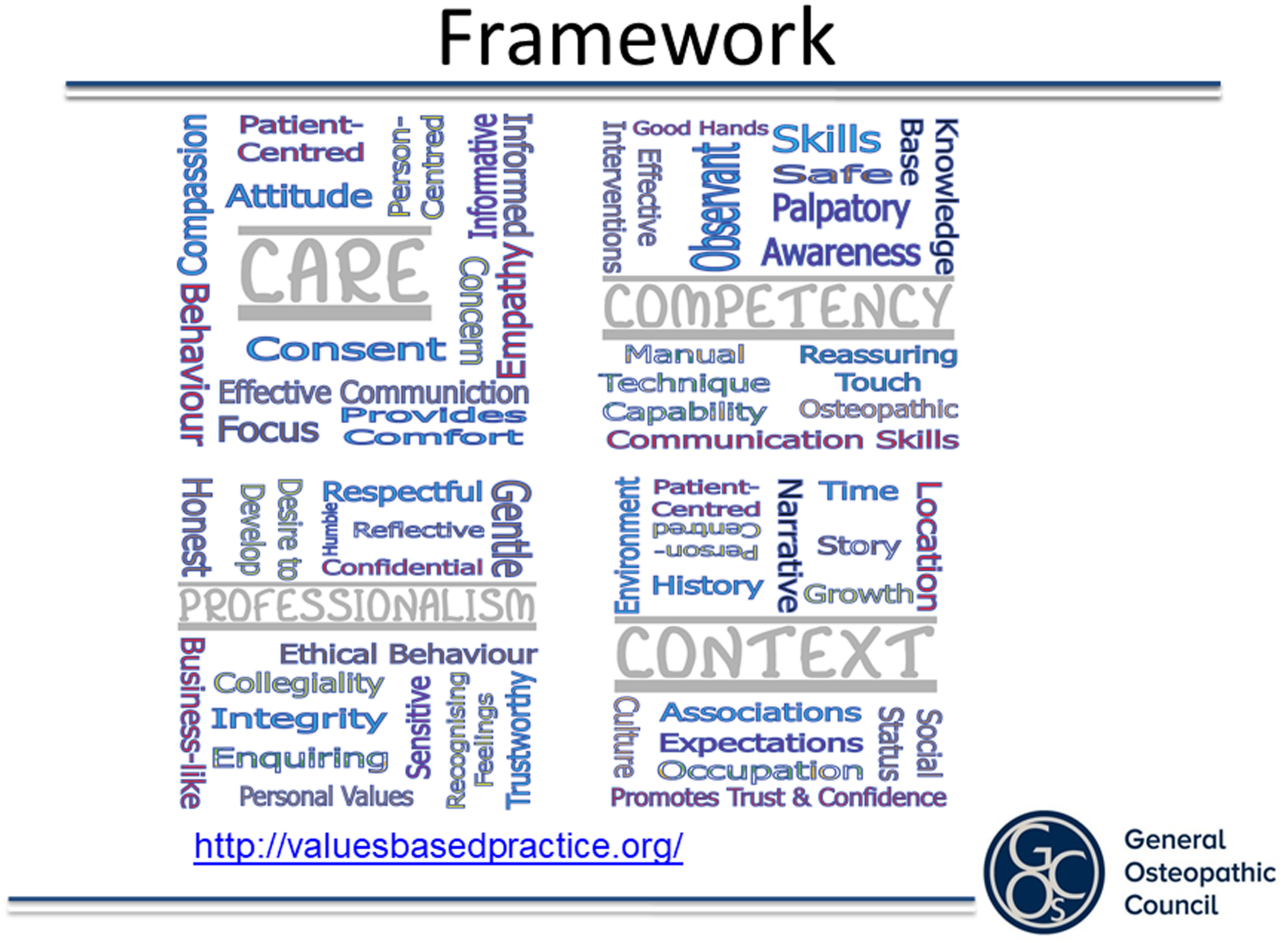
Common Core Values Framework

This framework is outlined in Figure 3.

### 2.2 Workshop 2

A second workshop was held on May 2015 at which the Common Core Values Framework (the framework) was used by participants, again including patients, osteopaths, and other health professionals, some of whom had attended the first workshop. The same method as for workshop 1 was used to explore case studies to see if the framework provided a method to support the identification of the values that might be important to patient and practitioner.

Various case studies were discussed with participants, applying the common framework to the case studies to gauge whether the framework provided an accurate model of the values that arose in the scenarios given and plenary feedback obtained and recorded.

The outcome was that the framework was not felt by all of the participants to provide a one‐size‐fits‐all approach to help practitioners identify what was important to patients. The framework was a broad and generic summary, but not necessarily something that could be easily applied in all circumstances. For example, some groups felt that the framework was reductionist in the area of competency, and others felt that key areas of practice including decision making, risk judgement, dealing with conflict of interest, and boundaries or roles, for example, did not fit naturally within the framework themes. It was felt that a different mechanism was required to help patients and practitioners identify what was important to them within the context of their osteopathic care.

### 2.3 Workshop 3

At this time, the GOsC began to work with the General Dental Council, which was also thinking about ways of effectively embedding its standards in professions where communication and consent were a common challenge.

Together, and in partnership with Community Research which was commissioned to support and report this work,^10^ the Collaborating Centre for Values‐based Practice, and the University College of Osteopathy, the steering group developed a “bottom up” method of engaging with patients and practitioners. This used patient and practitioner stories to better identify factors that enabled and produced barriers to the identification of what was important to patients in achieving a positive consultation.

This workshop took place in 2017 and involved patients and practitioners sharing their stories and dialogues. Patients were recruited by Community Research drawing on the patient groups of the GOsC and the General Dental Council as well as through a range of other methods to ensure a diverse range of patients. Characteristics of patients included a mix of gender, age, ethnicity, socio‐economic and work status. All patients had visited an osteopath or a dentist within the 6 months preceding the workshop. Practitioners were recruited via the professional communication networks of the GOsC with invitations available to all registrants. A series of mechanisms were in place to ensure the safety of patients and practitioners before, during, and after the workshops.

Following an introduction, participants took part in group discussions and an interactive “fruits, pests and roots” exercise. “The exercise was designed to uncover:

- The **fruits** or benefits of achieving a positive experience of a consultation.
- The **pests** or barriers to achieving it.
- The **roots** or actions that could be introduced to help overcome the barriers.

Using different coloured sticky notes (green/yellow for patients, red/pink/orange for practitioners) participants were asked to map the fruits, pests and roots onto a tree. Each table presented a summary of the actions during a plenary session and the pictures were pinned around the room for participants to view at leisure during a break.”

**Figure 4:**
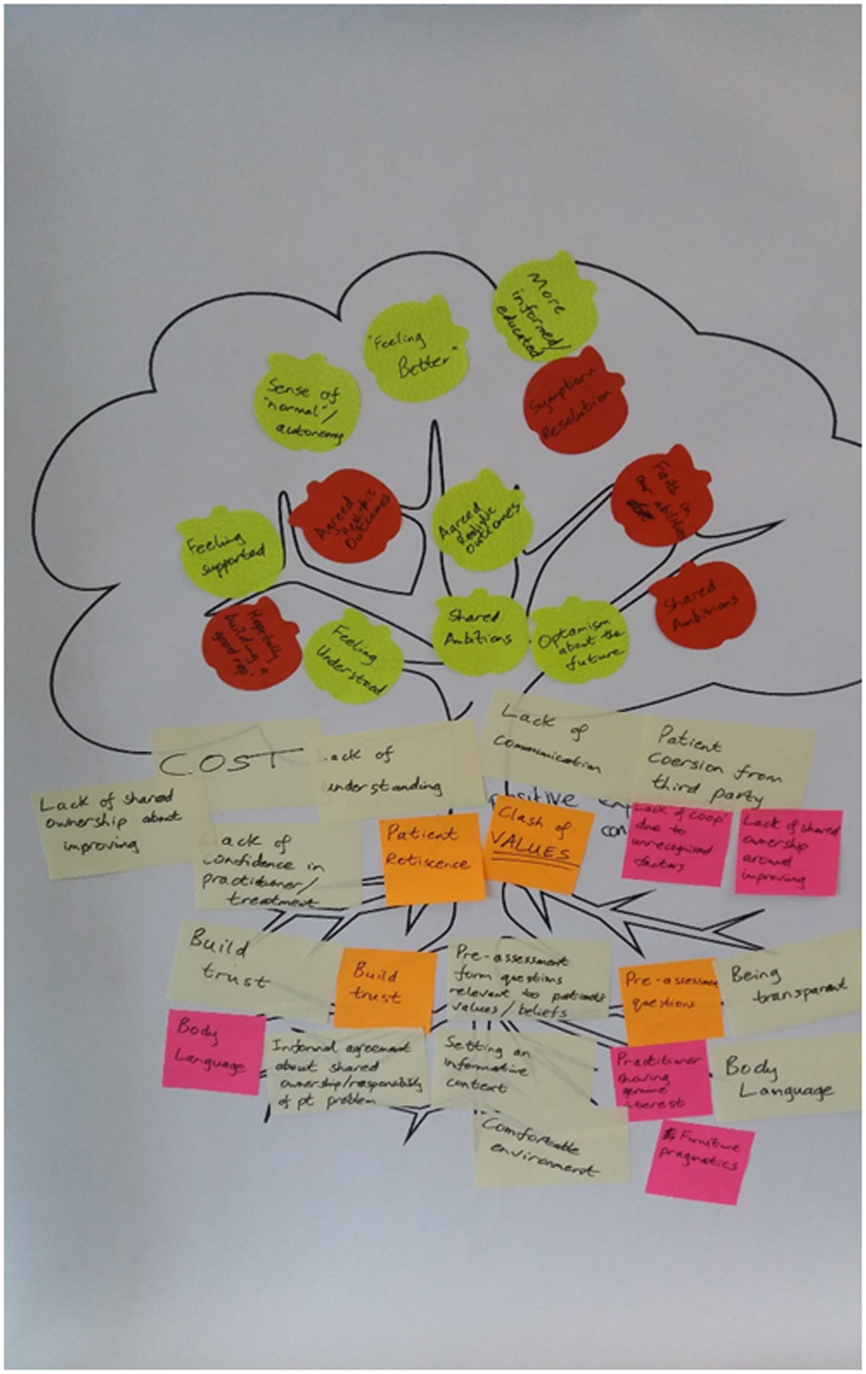
An example output from the fruits, pests and roots exercise

Figure 4 shows an example of one of the six trees developed by participants at the workshop.

“The final session of the workshop was dedicated to building questions that would be useful in establishing a patient's and practitioner's values within a consultation.”

Aspects contributing to a positive consultation identified by participants included the following:

- consultation starts at first point of contact;
- well‐presented reception and consultation space;
- clear communication of the options supported by visuals;
- the ability to listen/feeling listened to;
- sufficient time/not feeling rushed; and
- open and friendly demeanour.

Practitioners identified the following as important:

- patient taking ownership/responsibility (although note that not all patients wanted to do this);
- demonstrating empathy;
- remaining calm; and
- holistic approach

Patients identified the following as important:

- technical knowledge of practitioner and
- having confidence in practitioner.

Common emerging themes included

- taking a good patient history,
- developing a shared understanding/shared expectations,
- physical and logistical considerations,
- achieving good communication throughout the consultation, and
- reporting and acting on feedback,

but perhaps most importantly, the no “one‐size‐fits‐all” approach.

The outcome of the session was a rich series of data, stories, and themes, which enabled a core team comprising practitioners, patients, and communications experts to develop a series of approaches that might support more positive consultations.

## 3 DEVELOPING RESOURCES

Taking into account the workshop outcomes with the clear message that a single approach to identifying what was important to patients was not appropriate, the team developed a series of tools to support patients to prepare for consultations in a way that might best suit them. This work included developing resources aimed both at patients and at practitioners, to raise awareness of a values‐based approach to shared decision making and helping patients to consider and be more explicit about their expectations, preferences, concerns, and goals, consistent with the professional standards in place. These included the following.

### 3.1 Pilot resources for patients

Patient CV—this enables particularly patients with long‐term conditions to present their history in a way that is meaningful to them, not just their condition, but their life and what they do to support them to make clear to practitioners who they are and what they want and need.

http://www.osteopathy.org.uk/patient-cv-template/

Patient goal planner—this enables patients to identify their goals for their life (for example, picking up the children from school, doing the gardening, going swimming once a week, and being able to work without too much time off sick) and then to track over time how their symptoms or condition are affecting those goals.

http://www.osteopathy.org.uk/patient-goal-planner/

Infographic—leaflet or poster—this can be sent to the patient in advance to help them to think about their goals for the consultation, or it can be displayed in the reception area to help patients think about their goals whilst waiting to see the practitioner.

http://www.osteopathy.org.uk/values-infographic/

Animation—how to prepare for an osteopathic appointment

https://youtu.be/PC5jRadbv5M

### 3.2 Pilot resources for practitioners

Audio recording—listening to a discussion between Professor Bill Fulford and Professor Stephen Tyreman about values‐based practice

https://soundcloud.com/user-625548547/audio-interview-250918/s-CORFQ

Practitioner Reflection Sheet—enabling practitioners to rate their own perceptions of person‐centred care using the CARE measure

http://www.osteopathy.org.uk/practitioner-reflection-sheet/

## 4 DISCUSSION

Health professional regulation has mostly been known for its role in fitness to practise, which usually involves removing or restricting the right to practise of individuals, thus ensuring public protection. However, less attention is given by regulators to how standards are interpreted by a practitioner working in a particular context, to what health means to a patient, and how patients interact within their environment. And yet, it is in precisely this dialogue between patient and practitioner that regulatory standards are manifested.^11^

In 2018 and 2019, regulators are increasingly working towards “upstream” activity, embedding standards in a variety of ways to reduce instances of harm. This approach tends to focus on the provision of resources, guidance, advice, and dialogue, mostly for practitioners, to support the implementation of standards in collaboration with others in the sector, where relevant.^12^

Further, regulation research has shown that abstract rules can have unintended consequences. For example, perceptions of fitness to practise by registrants following cases that do not appear to have been dealt with fairly, according to their norms, can lead to greater disengagement from the regulator impacting on the patient safety that the regulator seeks to achieve.^13^

Whilst regulators may be able to demonstrate that awareness of standards is relatively high, regulators simply have registrant and patient generic perceptions. Regulators have little evidence about how the standards are actually manifested and applied and indeed the outcomes derived in the actual clinical setting.

Regulation has been challenged for increasing its interventions without evidence of their effectiveness.^14^ Research focussing on clinical practice and education has more recently highlighted the real‐world context within which health professionals work. Research shows correlations between fear and behaviour not in accordance with standards and anxiety leading to unethical behaviour, low trust, and low performance. Fulford's work on values‐based practice also shows that a focus on patient and clinician values can support contextual application of tools supporting decision making.^15^

In regulation, we are beginning to see evidence emerging of a more “relational” approach to regulatory activities. Communications and engagement strategies are given much more prominence, and we see evidence of regulators reaching outward rather than focussing inward. For example, McGivern's research^16^ shows engagement with registrants leading to reported better compliance with standards and opens the door to test out this approach empirically.

There is more evidence of regulators working together, responding in an agile way to challenges. Regulators are not just focussing on their statutory functions or processes.

In 2016, the legislation of all health professional regulators was changed to incorporate statutory objectives:

- to protect, promote, and maintain the health, safety, and well‐being of the public;
- to promote and maintain public confidence in the profession … ; and
- to promote and maintain proper professional standards and conduct for members of that profession.^17^

Potentially, this clear and comprehensive definition of the statutory objective of regulation provides a clearer space to support professionals to get to the right outcome (rather than focussing on doing the right things) and enables regulation to be a framework within which professionalism can flourish.

However, regulators are increasingly appreciating the complexity within which registrants work, and that they are not completely rational in their decision making, but human, with all that entails. In raising awareness and supporting implementation of these standards, regulators must think about the nature of humanity, and how humans make decisions.

Tyreman observed this some time ago noting that “Writing and policing policies to ensure ethical behaviour may be replacing the individual's integrity in acting ethically, where integrity performs the work of personal motivation, or ‘conscience’, in ensuring good behaviour.”^18^ In providing rules and regulations, regulators can inhibit the right decision being made because processes are being followed or guidance applied literally. In fact, we see this in all aspects of regulation, but because our perception of regulation, indeed, the very nature of the legal framework might be argued to be “about the right answer,” we pay less attention to the reality that the answer always involves the balancing of tensions; and the process of making that decision, and perhaps making this process explicit, becomes important in achieving the right outcome.

Regulation should be not necessarily doing things right in terms of processes but in terms of doing the right thing in terms of achieving the right outcome in any particular situation for the patient. Regulation should be a framework within which professionalism is able to flourish.^19^

Decision making, however, is not necessarily explicit or rational. There are a variety of ways of looking at this. But we know, for example, that culture and the environment represent shared ways of thinking, feeling, and behaving in health care organizations, and this is a contributing part of quality. “Those wishing and situated to improve services need a sophisticated understanding of the social dynamics and shared mental schema that underpin and reinforce existing practices and inform their readiness to change.”^20^ Mannion and Davies's systematic review shows us that whilst culture is not everything, it forms an important component informing decision making and that failing to take account of this can influence the delivery of policy, because the policy does not feature sufficiently in making the decision.

Potthoff and colleagues also undertook a systematic review about habit, and they said “Habit allows healthcare professionals to use their skills and training quickly and efficiently, minimising the cognitive load of active weighing of pros and cons in every clinical situation. However, when clinical guidelines of best practice change as new evidence and new interventions come to light, so too must behaviour.”^21^ Recognizing that much of decision making may not even be linked to reflective motivation, it happens almost automatically.

Further, in terms of creating the right environment for behaviour change, Michie et al suggested that to support behaviour change, behaviours must be precisely articulated. Once they are, creating the best conditions to support people to choose those behaviours requires thinking about capability, opportunity, and motivation (the COM‐B model). They said that “Behaviour is part of an interacting system involving all these components.”^22^ These components are as follows:

- Capability—ensuring that people have the physical skills, knowledge, cognitive and interpersonal skills, memory, attention and decision processes, and behavioural regulation—the right knowledge and skills and the ability to apply them to do the required behaviour change.
- Opportunity—ensuring that the environmental context and resources and the social influences around them create opportunities not barriers to undertake the required behaviour.
- Motivation—ensuring that the rational reasons for undertaking those behaviours are clear but also that the automatic motivation (the feel good) are stronger than those for undertaking alternative behaviours. Factors affecting motivation may include professional and social role or identity, beliefs about capabilities, optimism, goals, reinforcement, and emotion.

So supporting professionals (and patients) to make more explicit what is important for them may involve providing the knowledge and skills, and space and time to think about this and also that barriers to particular behaviours are removed. The dynamic interaction of the patient‐practitioner relationship also means that they both affect the environment, and a more prepared patient, able to articulate what is important to them, will necessarily impact on the behaviour of the practitioner, for example.

But motivation to understand the other, the patient, is potentially limited for a variety of reasons, including the experience and motivation of the practitioner.^23^

Anecdotal evidence has suggested a more positive patient experience that they share with others could provide a rational motivation for this work, but this would need to be field tested with the resources in the next stage of the project to refine and evidence this.

Health professional regulators refer to the concept of professional judgement often in their various standards but provide very little guidance about how to exercise this judgement. Regulators do not say that professional judgement is “what the professional thinks,” but perhaps that is how it is interpreted because there is no other guidance. Stephen Tyreman has written some insightful analyses of the concepts of integrity and whether this is a personally held moral framework or whether it is a social concept, which perhaps illustrates why it is difficult to define as part of a code of conduct because it is necessarily contextual and systemic and part of communities or meta‐values, which necessarily inform it.^24^ Another way of thinking about this is action‐based ethics (where good and bad decisions are judged by the act itself) and virtue ethics (where the focus is on the person). Petra Gelhaus has written on these points.^25^ Stephen Tyreman would argue that empathy and compassionate care should be developed within all health care professionals together with critical awareness and curiosity and openness plus evidence—all must be present for a good care.

When staff at the GOsC receive ethical queries, they cannot give the “right” answer, which is what professionals tend to want. This is because they cannot know the full context, or the detail of the views of the differing parties involved. But they can talk professionals through how they might make a decision or exercise their professional judgement in that scenario. If regulators were to provide guidance on the exercise of professional judgement, they might draw on a rational process of decision making, for example, drawn from the context of audit:

- asking the right question(s);
- evaluating evidence: quantitative and qualitative;
- looking from different perspectives (eg, patient, practitioner, society, and colleagues);
- seeking advice and alternative diverse perspectives to inform decision making; and
- deciding between options.

They must also be aware of the traps of decision making, for example:

- deciding too quickly;
- group think: the discussion fits with what I think already;
- overestimating our ability; and
- tendency to stick near to our original views.^26^

So far, we know that the process of health professionals making a decision may be influenced by a number of factors that may mitigate against taking into account the wants and needs of a patient.

But let us take a closer look at the patient who forms a central component of decision making. Indeed, without a patient decision made with informed consent, no intervention should take place.

Tyreman said “The practitioner's task is not merely to explain and treat, but to provide support and insight into the meaning of illness experiences in order to enable a patient to develop a better, life‐enhancing narrative and become a more whole person.”^27^ The argument is that health care is not about “fixing the patient” or “making them better,” but actually, it is about helping patients to live well in their environment, undertaking the daily activities that are important to them without being impeded by particular symptoms or a particular health condition.

In 2017, an article in *The Osteopath* magazine by Kenneth McLean reported the results of seeking patient feedback using the Care measure.^28^ The results reflected those of the Osteopathic Patient Expectations Research, showing 96% satisfaction with osteopathic treatment but also identified areas for improvement. The results also appear to mirror those seen in a revalidation pilot that took place in 2012, which also recorded slightly lower results in the areas, as follows:

“Patients generally scored practitioners very highly: on most questions, the ratings were either “very good” or “excellent”. Only three respondents gave a “good” rating to any questions (related to “**Really listening**” and “**Explaining things clearly**”), and two rated their practitioner as “fair” (on “**Helping you take control**” and “**Making a plan of action with you**” respectively). These responses gave us points to reflect on and be mindful of during future patient consultations.”

**Figure 5:**
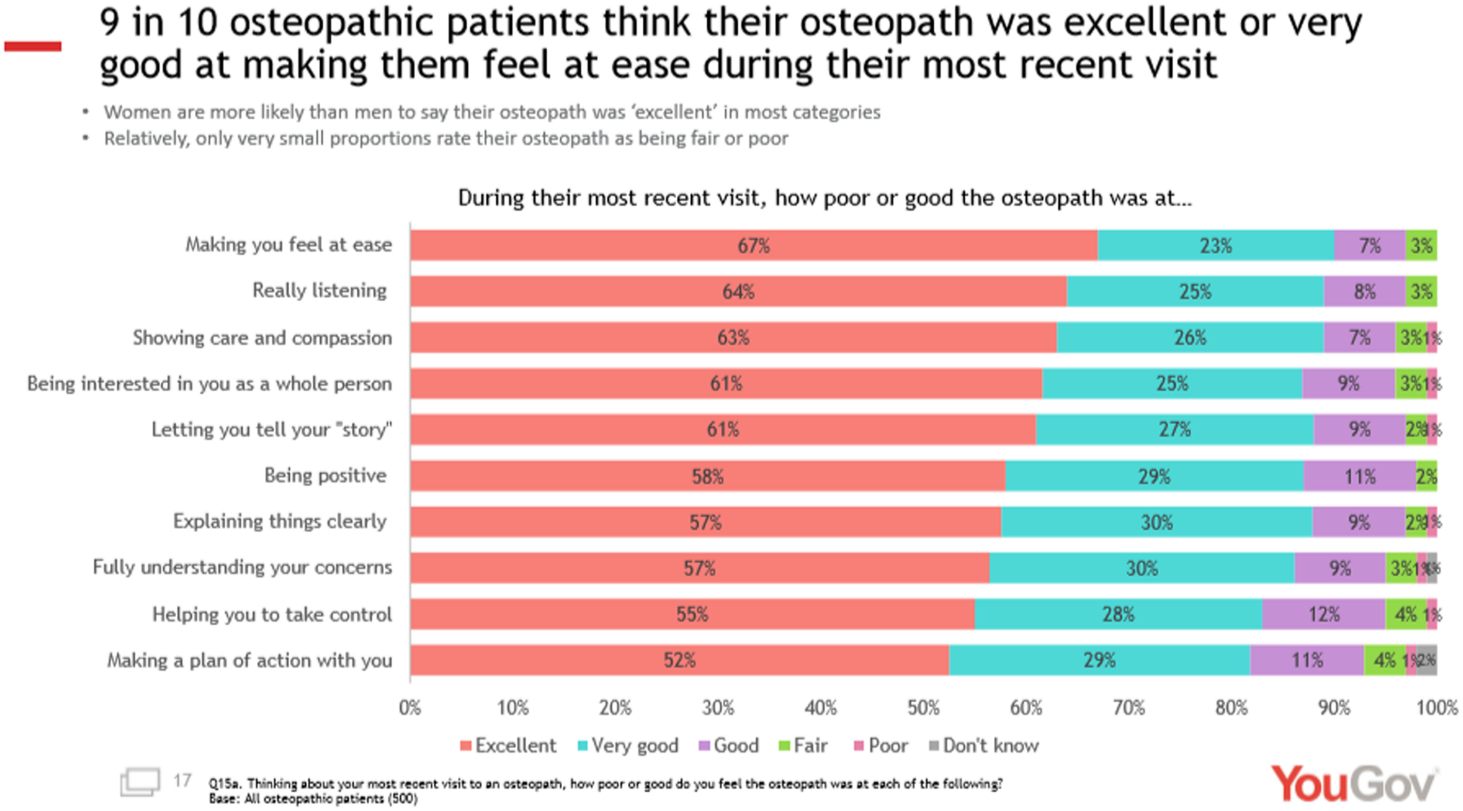
YouGov Survey, 2018

In 2018, the GOsC commissioned “YouGov” to seek feedback from a group of 500 patients who had had osteopathic treatment within the previous 6 months. Figure 5 shows^29^ the responses to the question “During their most recent visit, how poor or good was the osteopath at:

- Making you feel at ease
- Really listening
- Showing care and compassion
- Being interested in you as a whole person
- Letting you tell your ‘story’
- Being positive
- Explaining things clearly
- Fully understanding your concerns
- Helping you to take control
- Making a plan of action with you”

Again, these data show that areas such as “fully understanding your concerns,” “helping you to take control,” and “making a plan of action with you” were rated less highly suggestive of, perhaps, a paternalistic approach embedded into care.

**Figure 6:**
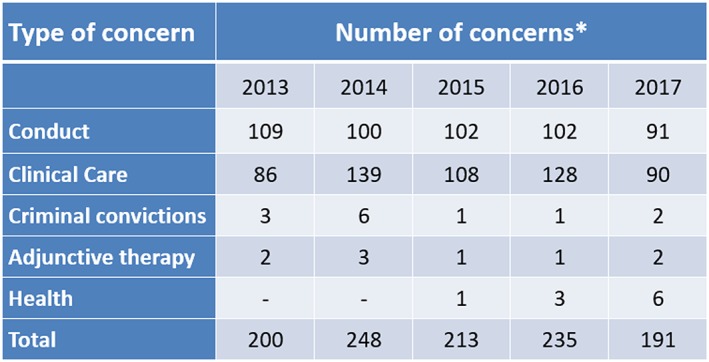
Summary data from the National Council for Osteopathic Research Report, 2018; types of concerns raised about osteopaths and osteopathic services in 2013 to 2017

The National Council of Osteopathic Research has been classifying first point of contact concerns each year received from patients reported to the regulator, to the professional indemnity insurers, and to the professional body (the Institute of Osteopathy) since 2013. Full data sets for 2013 to 2017 have been published,^30^ and a summary of the data is shown in Figure 6.

The data show that concerns around communication and consent feature in over half of patient‐reported concerns. In fact, even in clinical complaints, it is likely that communication challenges form a key part of those too.

In 2016 to 2018, the GOsC undertook a review of its core standards, the Osteopathic Practice Standards involving engagement and consultation with a range of stakeholders.^31^ Key changes included the following:

- updating communication and patient partnership and consent to ensure that patients were given the information that they “want and need” to better reflect the requirements for dialogue in the Supreme Court judgement of Montgomery;
- updating the section about boundaries—so that more guidance was provided about the role of the patient in defining boundaries including emotional and professional boundaries as well as sexual boundaries; and
- separating out the duty of candour from complaints so that candour is seen as a necessary part of good communication and not simply something that arises in the context of anticipated complaints and concerns.

Effective embedding of these subtle but important changes to our guidance requires further consideration of what happens in individual consultations and further thinking about how to support the embedding of our standards so that osteopaths think, feel, and behave in accordance with them, and they form an integral part of the decision‐making process.^32^

Our prototype resources have been developed ahead of piloting, which will test whether they have the potential to make a difference to patients and practitioners and whether they support patients and practitioners to make more explicit what is important to them in a consultation.

We plan to obtain feedback from patients and practitioners about the detail and design of each tool as well as the potential impact on practice through a pilot phase.

There are still some aspects of the feedback for which further tools or approaches need to be developed. In particular, the importance of the actual consultation environment was found to be particularly important to participants, but further work needs to be undertaken to enable this to be effectively translated into a tool for practitioners. We also need feedback about the tools from patients and practitioners who were not part of the development team.

The next phase of the project will use a range of methods including cluster sampling, pre‐testing and post‐testing with the CARE measure tool,^33^ and interviews and focus groups with users and practitioners to demonstrate impact.

Osteopaths work primarily in the independent sector, without teams, and usually practise outside the National Health Service (NHS) or managed environments. Much of the research starting to tread in this area is focussed on the NHS or managed environments—where there are many potential confounding factors in workplace culture.^34^ However, there are fewer confounding factors in place in osteopathy, which means that it is perhaps a better environment to test out these issues empirically.

### 4.1 What is the relevance for other health professions?

Many other health professionals also work outside the NHS or other managed environment governance structure, and so findings may be directly transferable to those. If the findings show that these tools or approaches are simple, cost‐effective, and easy to implement and results in improvements to patient feedback, they may support other health professionals within the NHS to achieve even better outcomes. There may also be some crossover between the commercial environment of independent practice and the resource‐limited environment of the NHS.

The resources developed to support clinicians to discern patient values and support a patient dialogue around consent could help to implement the legal changes to consent as outlined in the Montgomery judgement. This judgement provides that the information provided to the patient must be what is reasonable to that patient (through dialogue), rather than the previous Bolam test, which focussed on the information provided by a reasonable body of clinicians.^35^

We acknowledge, however, that it may be difficult to demonstrate a causative relationship between the use of the findings and potential changes to the CARE measure alone. We hope, though, that the qualitative work with patients and practitioners will help to assist us to more fully understand the factors contributing to or preventing a positive consultation.

## CONFLICT OF INTEREST

The General Osteopathic Council employed all the authors at the time of the study but without requiring a grant number.
